# Sero-Prevalence and Cross-Reactivity of Chikungunya Virus Specific Anti-E2EP3 Antibodies in Arbovirus-Infected Patients

**DOI:** 10.1371/journal.pntd.0003445

**Published:** 2015-01-08

**Authors:** Yiu-Wing Kam, Kwoon-Yong Pok, Kai Er Eng, Li-Kiang Tan, Simrandeep Kaur, Wendy W. L. Lee, Yee-Sin Leo, Lee-Ching Ng, Lisa F. P. Ng

**Affiliations:** 1 Singapore Immunology Network, Agency for Science, Technology and Research (A*STAR), Biopolis, Singapore; 2 Environmental Health Institute (EHI), National Environmental Agency, Singapore; 3 NUS Graduate School for Integrative Sciences and Engineering (NGS), National University of Singapore, Singapore; 4 Institute of Infectious Disease and Epidemiology (IIDE), Tan Tock Seng Hospital, Singapore; 5 Department of Biochemistry, Yong Loo Lin School of Medicine, National University of Singapore, Singapore; Centers for Disease Control and Prevention, United States of America

## Abstract

Chikungunya virus (CHIKV) and clinically-related arboviruses cause large epidemics with serious economic and social impact. As clinical symptoms of CHIKV infections are similar to several flavivirus infections, good detection methods to identify CHIKV infection are desired for improved treatment and clinical management. The strength of anti-E2EP3 antibody responses was explored in a longitudinal study on 38 CHIKV-infected patients. We compared their anti-E2EP3 responses with those of patients infected with non-CHIKV alphaviruses, or flaviviruses. E2EP3 cross-reactive samples from patients infected with non-CHIKV viruses were further analyzed with an *in vitro* CHIKV neutralization assay. CHIKV-specific anti-E2EP3 antibody responses were detected in 72% to 100% of patients. Serum samples from patients infected with other non-CHIKV alphaviruses were cross-reactive to E2EP3. Interestingly, some of these antibodies demonstrated clearly *in vitro* CHIKV neutralizing activity. Contrastingly, serum samples from flaviviruses-infected patients showed a low level of cross-reactivity against E2EP3. Using CHIKV E2EP3 as a serology marker not only allows early detection of CHIKV specific antibodies, but would also allow the differentiation between CHIKV infections and flavivirus infections with 93% accuracy, thereby allowing precise acute febrile diagnosis and improving clinical management in regions newly suffering from CHIKV outbreaks including the Americas.

## Author Summary

Chikungunya virus (CHIKV) causes Chikungunya fever in humans. The symptoms, particularly joint pain, can be severe and long lasting, and outbreaks can have serious socioeconomic impact. CHIKV is a mosquito-borne alphavirus that co-exists geographically with other mosquito-borne flaviviruses such as dengue virus (DENV). This causes difficulties in diagnosis because the symptoms are similar between CHIKV and DENV infections. It is important to differentiate between CHIKV and DENV infections, with good diagnostic methods. In this paper, we found that 72%–100% of CHIKV-infected patients had antibodies that recognized E2EP3, a part of a CHIKV protein. In contrast, a low percentage of flavivirus-infected patients had antibodies that recognized E2EP3. This suggests that testing patients for the presence of E2EP3-recognizing antibodies will aid in diagnostic differentiation between CHIKV and DENV infections. Interestingly, patients infected with non-chikungunya alphaviruses had moderate levels of antibodies that recognized E2EP3. While it was generally known that the alphaviruses have fairly conserved amino acid sequences, it was unknown until now, to what extent the antibodies against non-chikungunya viruses would also recognize E2EP3 from CHIKV. This paper provides insights about the E2EP3-recognizing antibodies from patients with different mosquito-borne viral infections and these insights will inform approaches to diagnostics and vaccination.

## Introduction

Chikungunya virus (CHIKV) has re-emerged as an important arbovirus that has caused unprecedented Chikungunya Fever (CHIKF) epidemics in Asia, Africa and more recently in the Americas [1]–[5]. Typical symptoms caused by CHIKV infection include fever, headache, myalgia, rash and debilitating arthralgia [6], [7]. These symptoms are largely similar to those caused by other arboviruses, especially the flaviviruses such as dengue virus (DENV) [8], [9]. In regions where DENV infections are endemic, there is also a likelihood of CHIKV infection as the two viruses share the common mosquito vectors *Aedes (Ae.) aegypti* and *Ae. albopictus*. Cases of CHIKV and DENV co-infection have been reported [10]–[13], surveillance systems in endemic regions have to be revised in order to manage this complicating situation. There is an increasing need for clinicians to differentiate between these two infections, and implement disease monitoring strategies in a timely manner.

Although simple indicators such as patient symptoms, or platelet count, may provide clues for distinguishing between CHIKV and DENV infections [14], these indicators do not lead to correct diagnosis with high enough accuracy. Thus, accurate and sensitive detection assays that can differentiate CHIKV from flavivirus infections are highly desired. Accurate diagnosis is important for epidemiological surveillance. It is also important for gaining a deeper understanding of the clinical manifestations of the different diseases. Ideally, a good diagnostic tool would be easy and quick to use, accurate, and sensitive. The current diagnostic tools available for CHIKV include PCR techniques to detect viral genomic material [15]–[17], and sero-diagnostic techniques identifying anti-CHIKV antibodies [18], [19]. PCR techniques are sufficient for diagnosis, but rely on the costly equipment and trained technical support. While antibody-based techniques are less costly and easier to handle, it is not known whether existing serological assays can detect CHIKV infection at early time points post illness onset.

The CHIKV E2 glycoprotein is one of the major targets of anti-CHIKV antibodies [20] and B-cell epitopes within the E2 glycoprotein have been identified previously in CHIKV-infected patients and relevant animal models [21]–[23]. One of the major B-cell epitopes, E2EP3, has been identified as the dominant B-cell epitope throughout the course of CHIKF disease in patients [20], [21]. Notably, the amino acid sequence within the E2EP3 is highly conserved across most CHIKV isolates, thus the presence of anti-E2EP3 IgG antibodies is potentially a good serological biomarker with useful implications for sero-epidemiology in populations of infection with different strains of CHIKV [5], [24]–[31].

Pre-clinical vaccination with E2EP3 has been shown to successfully reduce CHIKV-induced joint pathology in CHIKV challenged mice [20]. The E2EP3 domain is thus an important component in the development of CHIKV vaccines. To date, cross-reactivity between E2EP3 and antibodies obtained from non-CHIKV infected patients (e.g. patients infected with other alphaviruses, or with flaviviruses) has yet to be defined. Cross-reactivity would raise the possibility of developing cross-protective therapeutic strategies, which will especially benefit regions that experience epidemics caused by multiple closely-related arboviruses [32], [33].

To assess and characterize the level of cross-reactivity of anti-E2EP3 antibodies, retrospective serum samples were assayed from patients infected with CHIKV, non-CHIKV alphaviruses, DENV, and non-DENV flaviviruses. *In vitro* neutralization assays against CHIKV were also performed to establish the neutralizing capacities of these serum samples. Results demonstrated that 72% of CHIKV-infected patient samples exhibited detectable anti-E2EP3 antibody response, during the first 6 days post-illness onset (PIO). More than 95% of CHIKV-infected patients had detectable anti-E2EP3 antibody responses from 7 days PIO onwards who were screened across 1 day to 6 months PIO. Although the level of cross-reactivity among alphaviruses was more than 50%, only 6% of DENV-infected patients had antibodies that were cross-reactive to E2EP3. While antibodies from CHIKV-infected and some non-CHIKV alphavirus (Ross River virus or Barmah Forest virus)-infected serum samples neutralized CHIKV *in vitro*, none of the antibodies from flavivirus-infected serum samples did so. Therefore, this peptide-based ELISA assay not only serves as a sensitive method to accurately detect anti-CHIKV E2EP3 antibodies from CHIKV-infected patients, but also distinguishes CHIKV-infected patients from flavivirus-infected patients.

## Methods

### Ethics statement

Collection of CHIKV positive blood samples was approved by National Healthcare Group's Domain Specific Review Board (DSRB Reference No. B/08/026) as part of CDC's CHIKV Cohort study. Collection of anonymized residual sera after diagnostics testing from consented patient was approved by National Environment Agency (NEA) Bioethics Review Committee (IRB003.1) as part of EHI's Disease Surveillance and Diagnostics Development study. Written informed consent was obtained from all participants of both research studies. Usage of residual sera after diagnostics testing for evaluation of diagnostic assays to establish in-house capability is exempted from internal review by the NEA Bioethics Review Committee.

Cell lines such as baby hamster kidney (BHK21, ATCC CCL-10) cells were originally purchased from American Tissue Culture Collection (ATCC) and adhered to recommended ethics approvals and standards.

### Sample collection

Under the Chikungunya Cohort study, multiple consecutive samples were collected from Chikungunya confirmed (RT-PCR positive) patients referred to Communicable Disease Centre, Tan Tock Seng Hospital (CDC, TTSH). These suspected patients were tested positive for CHIK viral RNA [34] at Environmental Health Institute (EHI), the national reference laboratory for CHIKV during the 2008–2009 outbreak. Blood was collected during first medical consultation and subsequently, more samples were collected as the disease progressed, till convalescence and up to 6 months post-infection. All samples were kept at 4°C after phlebotomy, transported to EHI in cold-chain within 24 hours, processed and stored at −80°C. Two hundred and sixty samples from 38 CHIKV confirmed patients were selected to evaluate the sensitivity of the E2EP3 peptide ELISA. The post-illness onset (PIO) days of these selected samples range as early as 1 day till approximately 6 months post-illness onset.

Residual sera after diagnostics testing (at EHI) from 117 febrile patients who provided informed consent were selected to evaluate the specificity of E2EP3 peptide ELISA. These consented sera were anonymized and patient identifiers were removed before being used. Seventy-one were sera with unknown cause of infection and laboratory-confirmed negative for DENV and CHIKV antibodies. All 71 samples were screened using same set of tests for detection of dengue antibodies (IgM and IgG) and chikungunya antibodies (IgM and IgG). Panbio dengue IgM capture ELISA and Panbio dengue IgG Indirect ELISA (Alere) were used for detecting dengue IgM and IgG antibodies while anti-chikungunya IgM IIFT and IgG IIFT from EUROIMMUN AG were used for chikungunya IgM and IgG antibodies. The other 46 sera were laboratory-confirmed positive for DENV antibodies. In addition, residual sera from 14 non-DENV flavivirus antibody positive samples (4 yellow fever, 2 West Nile/Kunjin, 1 tick-borne encephalitis, and 7 non-specific flavivirus antibody positive samples) and 19 non-CHIKV alphavirus antibody positive samples (11 Ross River, 5 Barmah Forest, 1 sindbis and 2 Barmah Forest/Ross River antibody positive samples) sent for diagnostic testing were selected for evaluating assay's specificity. Arbovirus-specific antibody detection for these 33 samples were done by Public Health Virology, Queensland Health Forensic & Scientific Services, Australia using their in-house hemagglutination inhibition (HI) assay, genus- and virus-specific MAC-ELISA and IgG ELISAs. Typing for detection of antibodies against specific arbovirus is done using in-house IgM and IgG microsphere immunoassays (Luminex). All 33 samples were also tested against Anti-Chikungunya IgM and IgG IIFT developed by EUROIMMUN AG.

### Immunologic analyzes

Peptide-based ELISA was performed on clinical specimens as previously described [20], [21]. Biotinylated E2EP3 peptide was dissolved in dimethyl sulphoxide (DMSO) at a concentration of 15 µg/mL. Streptavidin-coated plates (Pierce) were first blocked with 1% sodium caseinate (Sigma-Aldrich) diluted in 0.1% PBST (0.1% Tween-20 in PBS), before coating with E2EP3 peptide diluted at 1∶1,000 in 0.1% PBST and incubated at room temperature for 1 h on a rotating platform. Plates were then rinsed with 0.1% PBST before incubation with serum samples diluted at 1∶4,000 with 0.1% PBST for 1 h. Plates were rinsed and then followed by incubation with goat anti-human IgG antibodies conjugated to HRP (Molecular Probes, 1∶4,000 dilution) diluted in 0.1% blocking buffer for 1 h at room temperature to detect peptide bound antibodies. Read-out was detected with TMB substrate solution (Sigma-Aldrich) and terminated with sulphuric acid (Sigma-Aldrich). Absorbance was measured at 450 nm in a microplate autoreader (Tecan). Clinical samples are considered E2EP3-positive if absorbance values are higher than the mean ±3 standard deviation (SD) values of healthy donor controls.

Commercial assay Anti-CHIKV IgG indirect immunofluorescence test (IIFT) from EUROIMMUN AG (Lübeck, Germany) was used according to manufacturers' instructions. Screening procedures were performed as described previously [27] except anti-human IgG fluorescein-conjugated secondary antibodies were used.

### Plaque Reduction Neutralization Technique (PRNT)

The level of cross-neutralizing activity to CHIKV was determined by PRNT assay using two local CHIKV strains (EHI0067Y08 and EHI1225Y08, isolated in 2008) [27]. The protocol was adapted from DENV PRNT as described [35]. Briefly, heat-inactivated sera was serially diluted at 1∶10, 1∶100 and 1∶1000 and incubated with an equal volume of CHIKV (800 plaque formation unit/ml) in 96-well plate for an hour at 37°C, 5% CO_2_. Fifty microliters of the virus-antibody mix was then added into 24-well plates containing Baby Hamster Kidney (BHK) cells in triplicates. The BHK cells were supplemented by MEM with 3% FBS, L-glutamine, sodium pyruvate and penicillin-streptomycin. The plates were incubated at 37°C and overlaid with Carboxy-Methyl-Cellulose (CMC) medium (containing MEM and supplements) within 16 hours. After two days of incubation at 37°C, cells were fixed with 20% formalin and stained using napthol blue staining solution. Plaques were counted and calculations of 50% end point plaque reduction neutralization titres were computed using log probit paper as described [36]. In this study, antibody titer above 1∶10 indicates presence of neutralizing activity to CHIKV.

### Statistics

All data are presented as mean ± SD. Differences in responses among groups were analyzed using appropriate tests (Mann-Whitney *U* tests, 2-sided Fisher exact test). A two-sided *P* value of less than 0.05 was considered to be statistically significant.

## Results

**Figure 1:**
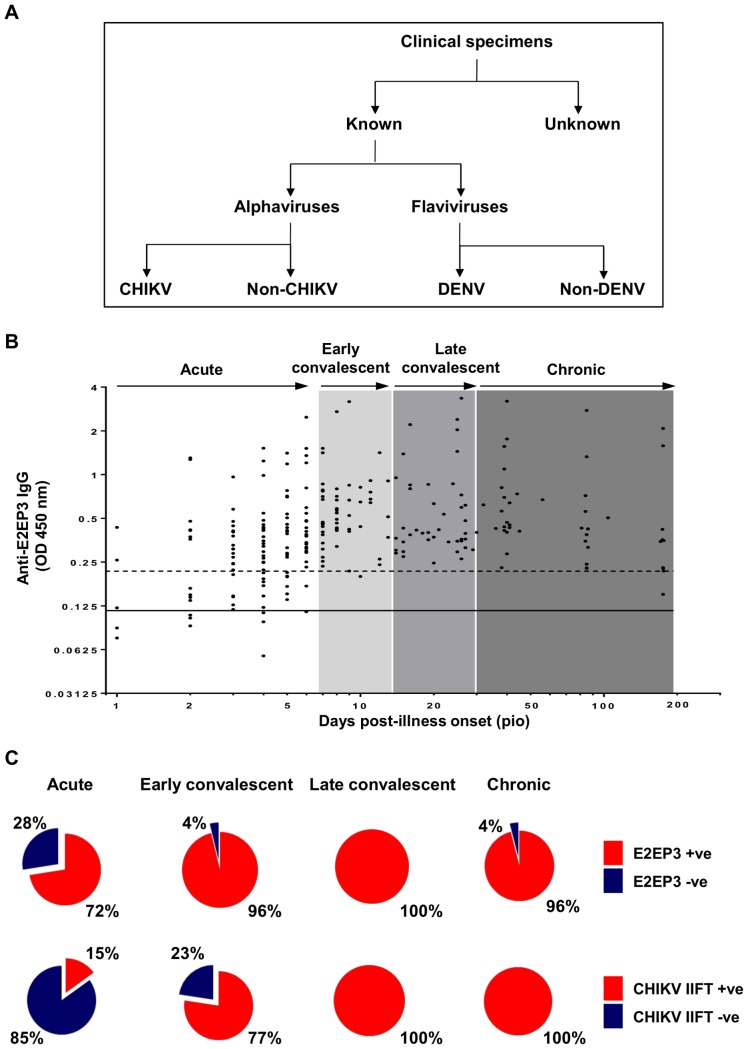
Antibody profiles of CHIKV-infected patients. *A,* Schematic diagram on the classification of clinical specimens. Four hundred and ten clinical specimens were included in this study, 339 samples have validated by in-house screening methods including 60 flaviviruses-positive samples and 279 alphaviruses-positive samples. Validated samples were further sub-categorized into 4 groups: CHIKV; Non-CHIKV (positive for alphavirus); DENV; and non-DENV (flavivirus-positive). Another 71 samples of febrile patients with unknown cause of infection were also included in this study. *B,* CHIKV E2EP3-specific IgG antibody detection in patients' serum samples (total 38 patients, n = 260) at a dilution of 1∶4000 were determined by peptide-based ELISA. Black solid line represents the mean value of the healthy donors and dotted line represents the value of mean ±3 SD. Values above mean ±3 SD are considered positive anti-E2EP3 IgG antibody response. Data are presented as mean. Patient samples were categorized into different phase including acute (1–6 days); early convalescent (7–13 days); late convalescent (14–29 days); chronic (30–176 days). *C*, Pie-chart shows the percentage of patients with positive or negative anti-E2EP3 IgG antibody response (upper panel), and the percentage of patients with positive or negative anti-CHIKV IgG response using immunofluorescence-based biochips (CHIKV IIFT, EUROIMMUN AG) (lower panel).

It was previously shown that E2EP3 is a dominant early serology marker in CHIKV-infected patient cohorts [20]. Here, we extend the study to another population cohort to investigate the sero-prevalence of anti-E2EP3 IgG antibodies in CHIKV-infected patients and also assess whether patients infected with other arboviruses (Fig. 1A) with similar clinical manifestations such as fever, myalgia, and arthralgia have cross-reactive antibodies against E2EP3.

Sero-prevalence of anti-E2EP3 IgG antibodies was performed in a peptide-based ELISA assay [20] in 38 individuals, as the level of E2EP3-specific IgM antibodies from CHIKV-infected patients during the early convalescent phase of disease (median 10 days pio) was very low (S1 Fig.). Therefore, anti-E2EP3 IgG antibodies were assessed in all subsequent studies. E2EP3-specific IgG antibodies were assessed in serum samples taken during the acute phase of 1–6 days PIO until the chronic phase of 30–176 days PIO (Fig. 1B). As expected, anti-E2EP3 IgG levels were observed to increase gradually from the acute to the early convalescent phase of 7–13 days PIO, and declined slightly during the late convalescent (14–29 days PIO) and chronic phases (Fig. 1B). Results showed that 72% of patient samples collected during the acute phase of disease showed E2EP3 sero-positivity (Fig. 1C, upper panel), while E2EP3 sero-positivity was more than 95% from the early convalescent phase to the later stage of disease progression (Fig. 1C, upper panel). This is similar to previous observations where significant increases in anti-CHIKV and anti-E2EP3 specific antibody responses were detected during the early convalescent phase of disease [20]. In contrast, the classical detection method using CHIKV-infected cell-based indirect immunofluorescence test (IIFT) could detect only 15% CHIKV positivity using the same set of acute-phase serum samples (Fig. 1C, lower panel). 100% CHIKV positivity was detected only during the late convalescent phase of disease (Fig. 1C, lower panel). This indicates the sensitivity difference between the two assays in CHIKV serology detection. Taken together, the peptide-based ELISA system serves as a more sensitive method than commercially available antibody-based assays, by detecting CHIKV anti-E2EP3 IgG antibodies during the acute phase of CHIKV infection.

**Figure 2:**
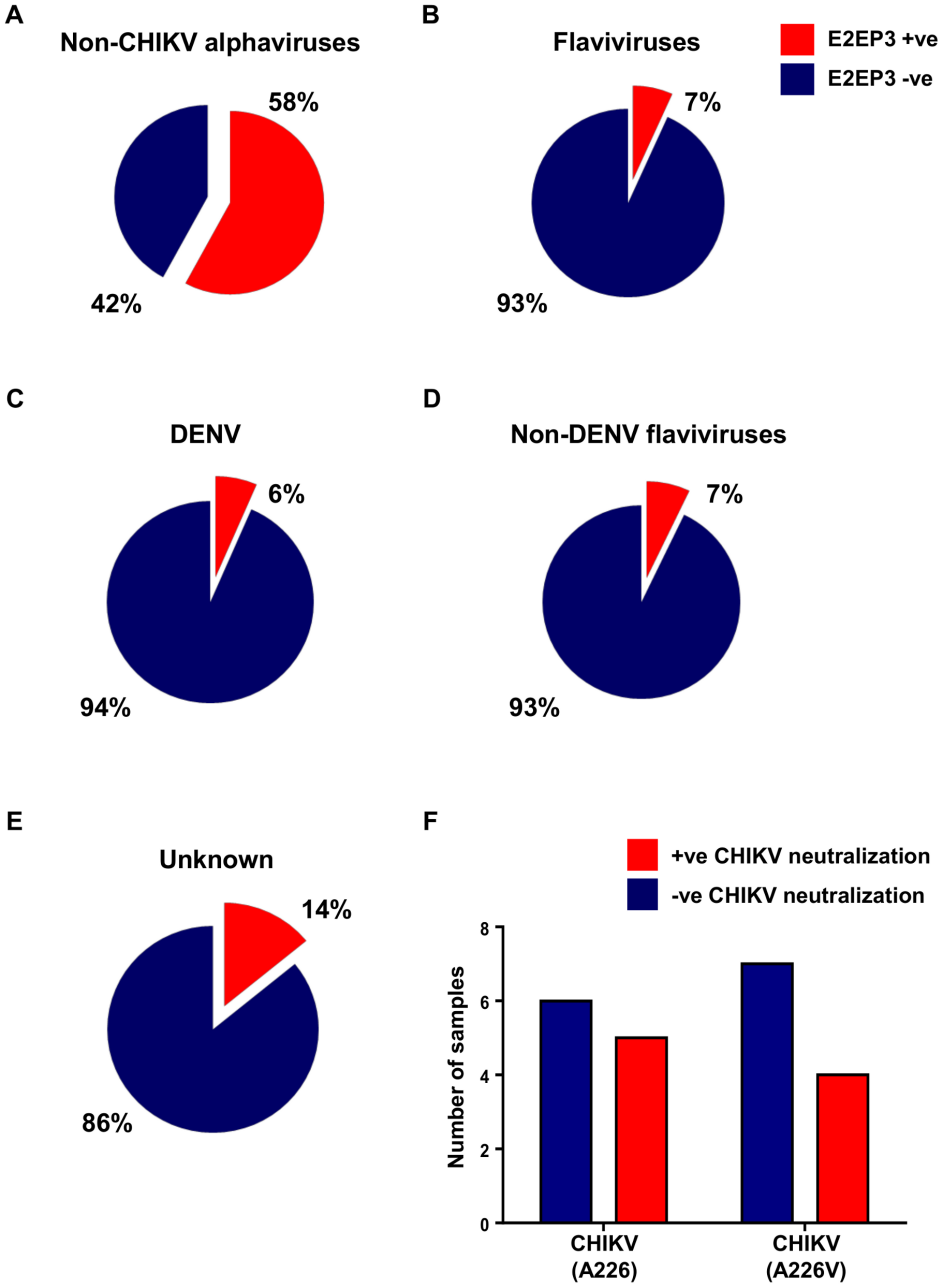
Cross-reactivity of anti-alphaviruses and anti-flaviviruses antibodies with CHIKV E2EP3. Patient serum samples caused by non-CHIKV alphaviruses infections (n = 19) and flaviviruses infections (n = 60) were screened for cross-reactivity against CHIKV E2EP3 by peptide-based ELISA at a dilution of 1∶4000. *A,* Pie-chart shows the percentage of non-CHIKV alphaviruses-infected patients samples with positive or negative anti-E2EP3 antibody response. *B,* Pie-chart shows the percentage of flaviviruses-infected patient samples with positive or negative anti-E2EP3 antibody response. *C - D,* Pie-chart shows the percentage of DENV-infected and non-DENV flaviviruses-infected patient samples with positive or negative anti-E2EP3 IgG antibody response respectively. *E,* Detection of CHIKV E2EP3 by serum samples of unknown infections. Serum samples (n = 71) of febrile patients from unknown cause of infections were screened by peptide-based ELISA at a dilution of 1∶4000. Pie-chart shows the percentage of patients' samples with positive or negative anti-E2EP3 antibody response. *F,* Bar-chart shows the number of non-CHIKV alphaviruses-infected, E2EP3 cross-reactive samples with and without CHIKV neutralizing activity against the two CHIKV isolates (CHIKV (A226) and CHIKV (A226V)).

To determine if the antibodies from other arbovirus-infected serum samples cross-reacted with E2PE3, peptide-based ELISA was further performed using samples from patients infected with non-CHIKV alphaviruses, or flaviviruses. Results revealed that patient serum samples infected with non-CHIKV alphaviruses, namely Ross River virus (RRV) and Barmah Forest virus (BFV), showed high level of cross-reactivity to E2EP3 (Fig. 2A). In addition, about 50% of these cross-reactive serum samples could neutralize CHIKV *in vitro* (Fig. 2F and Table 1).

**Table 1:** Neutralizing capacity of E2EP3 cross-reactive samples.

Patient	Type of infections	Anti-E2EP3 IgG (OD 450 nm)^a^	CHIKV (A226) Neutralization^b^	CHIKV (A226V) Neutralization^b^	IIFT^c^
1	CHIKV	0.725	86	>100	pos
2	CHIKV	0.432	60	>100	pos
3	CHIKV	0.270	58	66	pos
4	CHIKV	0.512	100	>100	pos
5	RRV	0.451	<10	48	equivocal
6	RRV	0.399	<10	16	equivocal
7	RRV	0.321	<10	<10	neg
8	RRV	1.187	<10	<10	equivocal
9	RRV	0.591	14	<10	equivocal
10	BFV	0.239	12	<10	neg
11	BFV	0.242	<10	15	equivocal
12	BFV	1.362	<10	<10	equivocal
13	RRV, BFV	1.238	35	<10	equivocal
14	RRV, BFV	0.821	28	<10	equivocal
15	RRV, DENV	0.447	36	19	neg
16	Flavivirus	0.246	<10	<10	neg
17	DENV	0.267	<10	<10	neg
18	DENV	0.234	<10	<10	neg
19	DENV	0.283	<10	<10	neg
20	Unknown	0.248	<10	<10	neg
21	Unknown	0.570	<10	<10	neg
22	Unknown	0.260	<10	<10	neg
23	Unknown	0.262	<10	<10	neg
24	Unknown	0.236	<10	<10	neg
25	Unknown	0.248	<10	<10	neg
26	Unknown	0.248	<10	<10	neg
27	Unknown	0.243	<10	<10	neg
28	Unknown	0.269	<10	<10	neg
29	Unknown	0.229	<10	<10	neg

a Anti-E2EP3 IgG antibody titer was determined by peptide-based ELISA from serum samples with different type of infections. O.D. values > 0.216 were classified as E2EP3 cross-reactive samples.

b Antibody titer above 1∶10 indicates presence of neutralizing activity to CHIKV.

c CHIKV detection was considered as equivocal if the weak immunofluorescence was observed from the BIOCHIP.

Interestingly, a low level of cross-reactivity (7%) was observed when patient serum samples infected with flaviviruses such as DENV, yellow fever virus (YFV), West Nile virus (WNV), Kunjin virus (KUNV), and tick-borne encephalitis virus (TBEV) were screened against E2EP3 (Fig. 2B). Specifically, there was a 6% of cross-reactivity from DENV-infected patient serum samples (Fig. 2C). Similar observations were obtained from non-DENV flavivirus-infected patient serum samples (Fig. 2D). Overall, Fig. 2B–D suggests negligible differences in cross-reactivity against E2EP3, between samples infected by DENV, and samples infected by non-DENV flaviviruses.

In addition, 14% of serum samples obtained from febrile patients with unknown causes of infection showed sero-positivity against E2EP3 (Fig. 2E). However, none of these serum samples demonstrated any CHIKV *in vitro* neutralizing activity. This could be due to the presence of low levels of anti-E2EP3 IgG antibodies. While a previous study has detected long-lasting anti-E2EP3 IgG antibody responses in patients 2 years post CHIKV infection [21], declining levels of anti-E2EP3 IgG antibodies may still contribute to the lack of neutralizing activity in *in vitro* neutralization assays.

**Figure 3:**
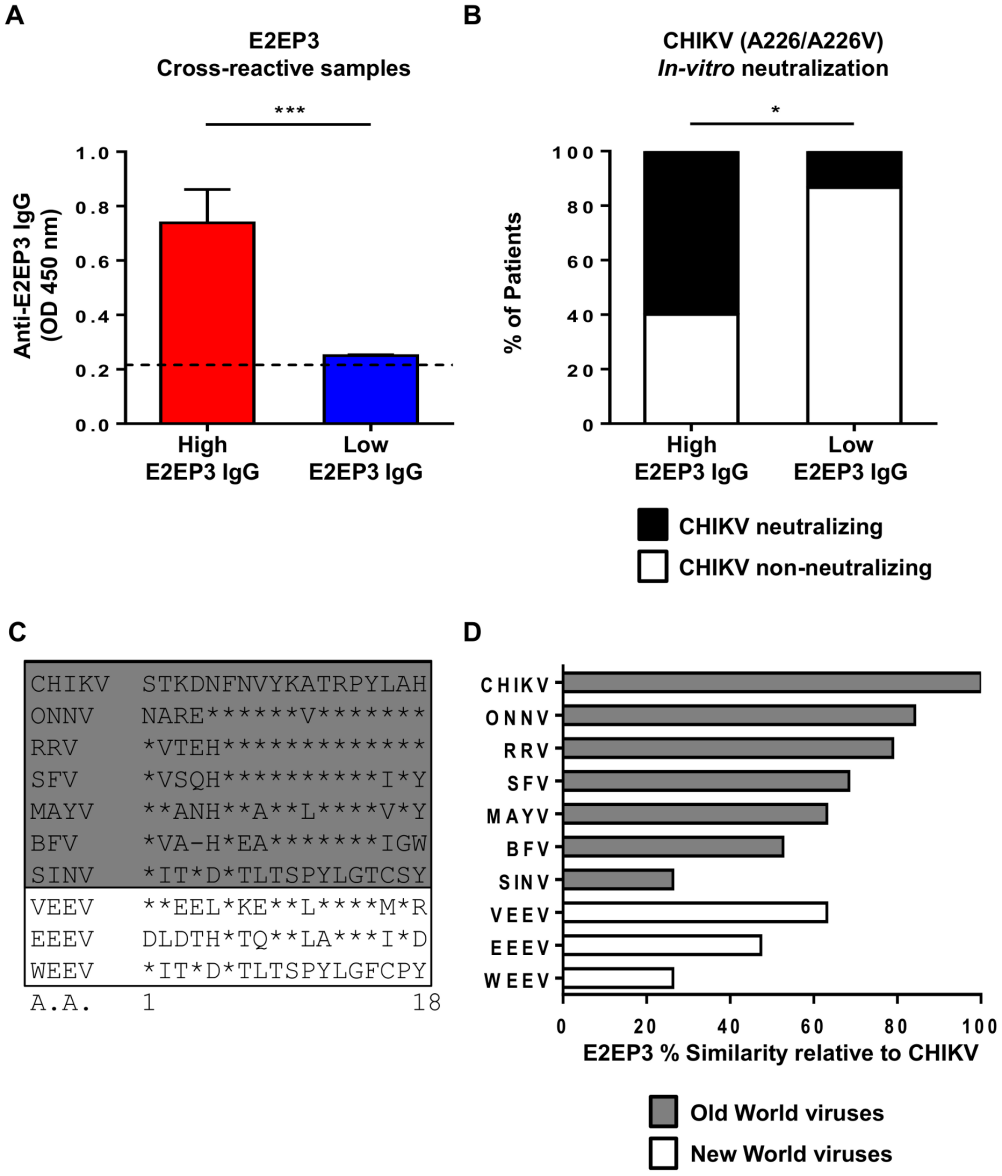
Association of E2EP3 cross-reactive antibody responses with CHIKV neutralizing activity. *A,* Anti-E2EP3 antibody response in serum samples (High E2EP3 IgG responders, n = 10; Low E2EP3 IgG responders, n = 15), at a dilution of 1∶4,000 were determined by ELISA using biotinylated E2EP3 peptides. Data are presented as mean ± SEM. Data are representative of two independent experiments with similar results. Statistical significance was measured using Mann-Whitney *U* test (***P<0.0001). *B, In-vitro* neutralizing activity against CHIKV in High and Low E2EP3 IgG responders. Histogram shows the percentage of patient samples with or without CHIKV neutralizing activity. Statistical significant was measured using 2-sided Fisher exact test between the patient samples with and without CHIKV neutralizing activity in the 2 responder groups. *P = 0.028. *C - D,* E2EP3 sequence from CHIKV was aligned to corresponding sequences from 9 non-CHIKV alphaviruses (ONNV, RRV, SFV, MAYV, BFV, SINV, VEEV, EEEV and WEEV). Conserved amino acid (A.A.) are indicated by a *. Percentage similarity between CHIKV E2EP3 sequence and corresponding sequence from each non-CHIKV alphavirus were calculated with SIAS (http://imed.med.ucm.es/Tools/sias.html). Percentage similarity relative to CHIKV E2EP3 is indicated by a bar-chart. Viruses classified as Old World alphaviruses are marked by the grey shading box, while viruses classified as New World alphaviruses are marked by the white box. NCBI Genbank accession numbers of ten selected alphaviruses (CHIKV - DQ443544, ONNV - O90369, RRV - AEC49728, SFV - ABA29033, MAYV - AAL79764, BFV - NP819000, SINV - ACU25462, VEEV - AAB02519, EEEV - NP740646, WEEV - NP818940).

To further understand the relationship between anti-E2EP3 IgG antibody levels and *in vitro* CHIKV neutralization, E2EP3 cross-reactive samples (including those infected with non-CHIKV alphaviruses, flaviviruses, and unknown sources of infection) were separated into high and low anti-E2EP3 IgG groups based on E2EP3 peptide-based ELISA (categorizing based on the median value) (Fig. 3A). In the high anti-E2EP3 IgG group, 60% of samples neutralized CHIKV *in vitro*, while less than 20% did so in the low anti-E2EP3 IgG group (Fig. 3B). These results suggest an association between high levels of E2EP3 cross-reactive antibodies, and *in vitro* CHIKV neutralization.

The high E2EP3 cross-reactivity found in non-CHIKV alphavirus-infected patient samples (Fig. 2A) could be due to a previous CHIKV infection and/or the sequence similarity of the E2EP3 region between CHIKV and non-CHIKV alphaviruses. The E2EP3 amino acid sequence from CHIKV was aligned to corresponding sequences from 9 non-CHIKV alphaviruses and amino acid similarity levels were compared (Fig. 3C and 3D). Most of the Old World non-CHIKV alphaviruses (excluding SINV) exhibited more than 50% sequence similarity relative to CHIKV E2EP3 and this could explain the observation that non-CHIKV alphaviruses (e.g. RRV and BFV) induced antibodies that recognized the CHIKV E2EP3 sequence in the peptide-based ELISA system. In contrast, New World non-CHIKV alphaviruses exhibited low levels of sequence similarity relative to CHIKV E2EP3. Low levels of antibody cross-reactivity against E2EP3 and lack of CHIKV neutralizing capacity found in flaviviruses-infected samples (Fig. 2B and Table 1), are consistent with the greater sequence diversity between alphaviruses and flaviviruses [37]

## Discussion

In this study, the sero-prevalence of anti-E2EP3 IgG antibodies in patients infected with CHIKV as well as non-CHIKV viruses was analyzed in detail. Anti-E2EP3 IgG antibodies were detectable by ELISA as early as 1 day PIO in CHIKV-infected patients. Moreover, it was demonstrated that in comparison with the existing indirect immunofluorescence test (IIFT) for CHIKV detection, the E2EP3 peptide-based ELISA was able to detect anti-CHIKV antibodies in a higher percentage of patient samples taken during the acute and early convalescent phases of disease. More than 95% sero-prevalence of anti-E2EP3 IgG antibodies from as early as 6 days PIO was detected with the E2EP3 peptide-based ELISA. The timing of sample collection affects the detectability of antibody responses to CHIKV infection. Here, detection of anti-E2EP3 IgG antibodies would be a good early serology detection approach for samples taken from 6 days up to 29 days PIO.

The sensitivity of serologic diagnostic assays varied between samples obtained from patients infected with different isolates of CHIKV (CHIKV-A226 or CHIKV-A226V) [27]. The assays were based on detection of antibodies against whole virus-based antigens, and mutations in the E1 or E2 glycoprotein were demonstrated to be the cause of the differences in assay sensitivity. In contrast, since E2EP3 is a highly conserved region within the E2 glycoprotein, the detection of anti-E2EP3 antibodies is expected to be largely similar in terms of sensitivity for different CHIKV isolates.

The detection of anti-E2EP3 IgG antibodies in up to >95% of samples from CHIKV-infected patients is striking, considering that only 20% to 80% of patients were seropositive for antibodies against viral antigens in other studies [38]–[41]. The 100% sero-prevalence of anti-E2EP3 IgG responses in late-convalescent CHIKV patients could be due to E2EP3 immunodominance, and/or pre-existing immunity against CHIKV in the population. Pre-existing immunity against CHIKV could be due to asymptomatic CHIKV infection [42], or the presence of natural antibodies [43]. Asymptomatic virus infection induces pre-existing antibodies of which the levels could be age-dependent [41]. The existence of natural antibodies has been previously proposed in mice and humans [44]. These antibodies could be an important component in immunity as they provide protection during very early stages of infection [43]. Moreover, secretion of pre-immune poly-reactive IgG from un-stimulated B cells could establish an *in vivo* protective response with components from the innate immune system [45]. While it is known that natural antibodies in mouse sera could recognize CHIKV antigens and neutralize CHIKV *in vitro* [22], whether and to what extent natural antibodies against CHIKV exist in human populations is still unclear.

Cross-reactivity of E2EP3 observed in the non-CHIKV alphavirus-infected samples (Fig. 2A) raises the question of whether cross-protection or cross-neutralization can occur in cases where a patient is infected by different, but closely related alphaviruses, particularly when two different alphaviruses circulate within the same geographic region [33], [46]. Serum samples from non-CHIKV alphavirus-infected patients contained antibodies that detected E2EP3, and interestingly, some of these antibodies could even neutralize CHIKV *in vitro*. While it could be that the cross-reactive antibodies were also cross-neutralizing due to the high sequence similarity amongst alphaviruses (Fig. 3D). Physicochemical property of an epitope such as the isoelectric point of the E2EP3 peptide region from CHIKV and non-CHIKV alphaviruses may contribute to cross-reactivity in the ELISA assay, as well as cross-neutralization *in vitro*. However, one cannot exclude the possibility of previously undetected CHIKV co-infections or asymptomatic CHIKV infections in the non-CHIKV alphavirus-infected patients [42], as the cause of the CHIKV neutralizing capacity of their serum. High levels of anti-E2EP3 or E2EP3 cross-reactive antibodies correlated with CHIKV neutralizing capacity of the serum samples in this study. However, it leaves to be uncovered whether the neutralizing capacity is due to E2EP3 cross-reactive antibodies, or bona fide anti-E2EP3 antibodies from unreported CHIKV co-infections.

Although 7% of flavivirus-infected patient samples contained antibodies recognizing E2EP3, none of these serum samples neutralized CHIKV *in vitro*. The lack of CHIKV neutralizing ability in the flavivirus-infected serum samples suggests that although CHIKV and flavivirus co-infections have been reported [13], the likelihood of unreported CHIKV co-infection within this flavivirus-infected group is low. The E2EP3 ELISA thus has an error of 7%, mis-identifying flavivirus infection as potentially CHIKV infection. However, this error rate is comparable with pre-existing commercial CHIKV diagnostics, for example the NovaLisa ELISA for CHIKV antigen-specific IgG has a reported specificity >90% [47].

The E2EP3 peptide-based ELISA can also be used to track antibody status after CHIKV infection. Such immune monitoring approaches can form the basis for understanding clinical outcomes. Antibody-based assays have been demonstrated to predict wide variation of clinical manifestations among patients infected with the same viral strain such as in respiratory syncytial virus [48], and Hepatitis C virus [49] infection studies. The links between CHIKV viral load, IgG3 and clinical consequences [50] have been defined, where the anti-CHIKV IgG3 was composed largely of anti-E2EP3 IgG antibodies [20]. Here, anti-E2EP3 IgG antibodies were validated to be a good early serology marker for CHIKV-infected patients. Thus, the potential in using the E2EP3 peptide-based ELISA for immune monitoring in CHIKV infection will be a strong advantage compared to CHIKV genome PCR, which provides for only early diagnosis but does not relay information about the immune status of the patients. The quantitative measure of anti-E2EP3 IgG antibody detection provided by the peptide-based ELISA assay can also be useful in future clinical applications and disease management, in comparison to assays which only provide a yes/no diagnostic readout. In regions where CHIKV is co-endemic with DENV, detection of anti-E2EP3 IgG antibodies will help clinicians distinguish between CHIKV and DENV infection at early stages of disease, which would allow specific treatments to be provided promptly, thereby optimizing clinical management. Potentially, it could be a useful tool to enable accurate and rapid diagnosis for public health agencies in the current explosive outbreaks of painful CHIKF in the Caribbean islands.

## Supporting Information

S1 Fig **Anti-E2EP3 IgG and IgM response from CHIKV-infected patient samples.** CHIKV-infected patient (Singapore cohort 2008–2009, Kam *et al*., 2012 J Infect Dis) plasma pools (30 patient samples) collected at median 10 days pio were subjected to E2EP3 peptide-based ELISA at a dilution of 1∶2000, followed by anti-human-IgG HRP-conjugated (1∶4000) and anti-human-IgM HRP-conjugated (1∶4000) secondary antibodies. *** *P*<0.0001 by Mann–Whitney *U* test. Experiments were performed in duplicates.
(TIF)

S1 Checklist **STARD checklist.**
(DOC)
