# Supplementary figures and images for: Sero-Prevalence and Cross-Reactivity of Chikungunya Virus Specific Anti-E2EP3 Antibodies in Arbovirus-Infected Patients
Source: PLoS Negl Trop Dis. 2015 Jan 8;9(1):e3445. doi: 10.1371/journal.pntd.0003445 (PMC4287563; doi:10.1371/journal.pntd.0003445)

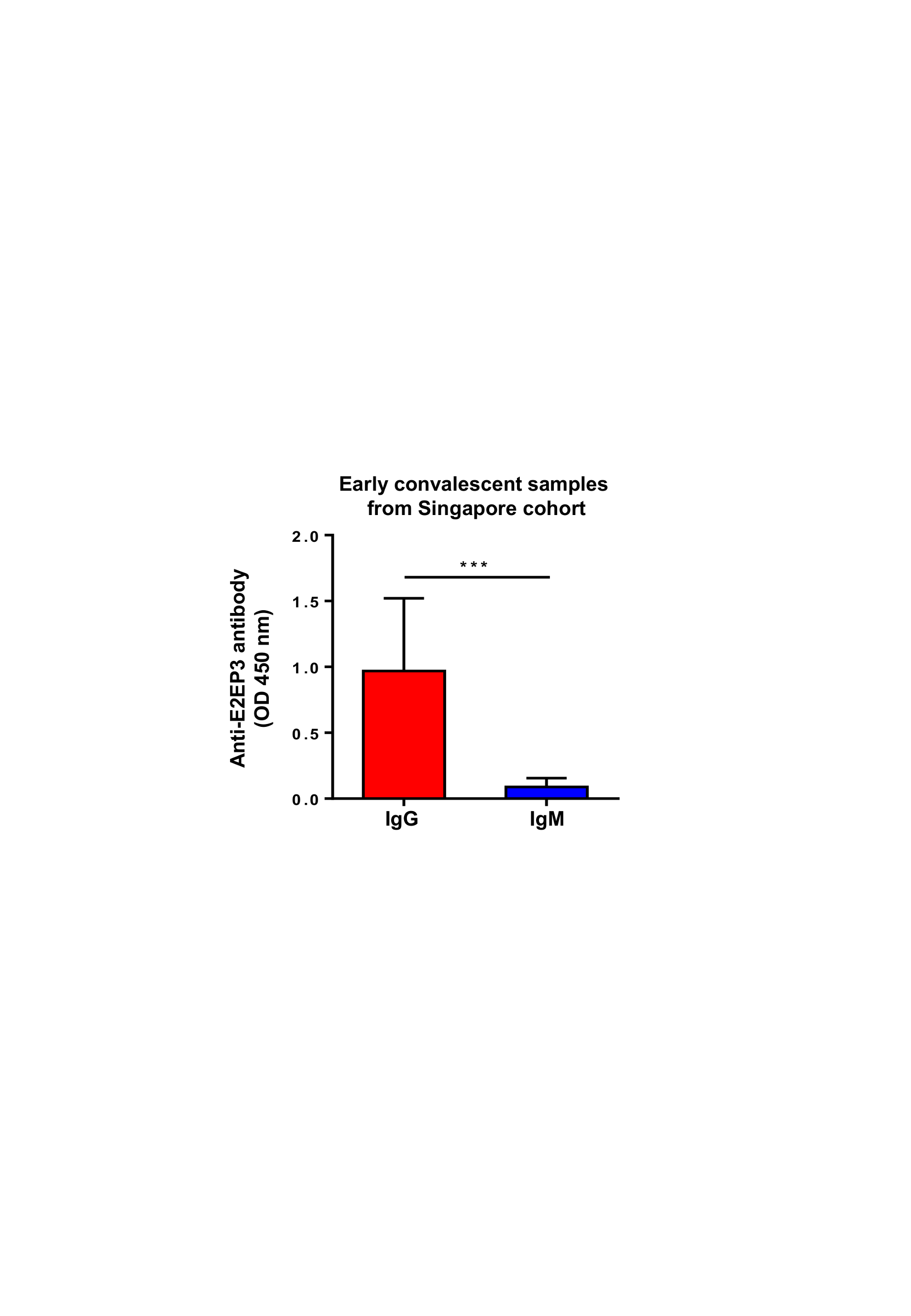

Supplement: S1 Fig — Anti-E2EP3 IgG and IgM response from CHIKV-infected patient samples. CHIKV-infected patient (Singapore cohort 2008–2009, Kam et al., 2012 J Infect Dis) plasma pools (30 patient samples) collected at median 10 days pio were subjected to E2EP3 peptide-based ELISA at a dilution of 1∶2000, followed by anti-human-IgG HRP-conjugated (1∶4000) and anti-human-IgM HRP-conjugated (1∶4000) secondary antibodies. *** P<0.0001 by Mann–Whitney U test. Experiments were performed in duplicates. (TIF) [file pntd.0003445.s001.tif]
